# Potential for Virus Endogenization in Humans through Testicular Germ Cell Infection: the Case of HIV

**DOI:** 10.1128/JVI.01145-20

**Published:** 2020-11-23

**Authors:** Dominique Mahé, Giulia Matusali, Claire Deleage, Raquel L. L. S. Alvarenga, Anne-Pascale Satie, Amélie Pagliuzza, Romain Mathieu, Sylvain Lavoué, Bernard Jégou, Luiz R. de França, Nicolas Chomont, Laurent Houzet, Antoine D. Rolland, Nathalie Dejucq-Rainsford

**Affiliations:** a Université Rennes, INSERM, EHESP, IRSET (Institut de Recherche en Santé, Environnement et Travail)—UMR_S1085, Rennes, France; b Laboratory of Cellular Biology, Department of Morphology, Federal University of Minas Gerais, Belo Horizonte, Brazil; c Department of Microbiology, Infectiology and Immunology, Faculty of Medecine, Université de Montréal, and Centre de Recherche du CHUM, Montréal, Quebec, Canada; d Centre Hospitalier Universitaire de Pontchaillou, Service Urologie, Rennes, France; e Centre Hospitalier Universitaire de Pontchaillou, Centre de Coordination des Prélèvements, Rennes, France; Ulm University Medical Center

**Keywords:** virus, HIV, SIV, testis, spermatogenesis, gametes, entry, integration, replication, endogenization, male germ cells, restriction factors, antiviral defense, cell-associated infection, evolution

## Abstract

Viruses have colonized the germ line of our ancestors on several occasions during evolution, leading to the integration in the human genome of viral sequences from over 30 retroviral groups and a few nonretroviruses. Among the recently emerged viruses infecting humans, several target the testis (e.g., human immunodeficiency virus [HIV], Zika virus, and Ebola virus). Here, we aimed to investigate whether human testicular germ cells (TGCs) can support integration by HIV, a contemporary retrovirus that started to spread in the human population during the last century. We report that albeit alternative receptors enabled HIV-1 binding to TGCs, HIV virions failed to infect TGCs *in vitro*. Nevertheless, exposure of TGCs to infected lymphocytes, naturally present in the testis from HIV^+^ men, led to HIV-1 entry, integration, and early protein expression. Similarly, cell-associated infection or bypassing viral entry led to HIV-1 integration in a spermatogonial cell line. Using DNAscope, HIV-1 and simian immunodeficiency virus (SIV) DNA were detected within a few TGCs in the testis from one infected patient, one rhesus macaque, and one African green monkey *in vivo*. Molecular landscape analysis revealed that early TGCs were enriched in HIV early cofactors up to integration and had overall low antiviral defenses compared with testicular macrophages and Sertoli cells. In conclusion, our study reveals that TGCs can support the entry and integration of HIV upon cell-associated infection. This could represent a way for this contemporary virus to integrate into our germ line and become endogenous in the future, as happened during human evolution for a number of viruses.

**IMPORTANCE** Viruses have colonized the host germ line on many occasions during evolution to eventually become endogenous. Here, we aimed at investigating whether human testicular germ cells (TGCs) can support such viral invasion by studying HIV interactions with TGCs *in vitro*. Our results indicate that isolated primary TGCs express alternative HIV-1 receptors, allowing virion binding but not entry. However, HIV-1 entered and integrated into TGCs upon cell-associated infection and produced low levels of viral proteins. *In vivo*, HIV-1 and SIV DNA was detected in a few TGCs. Molecular landscape analysis showed that TGCs have overall weak antiviral defenses. Altogether, our results indicate that human TGCs can support HIV-1 early replication, including integration, suggesting potential for endogenization in future generations.

## Abstract (precis)

Viruses have colonized the host germ line on many occasions during evolution to eventually become endogenous. Here, we aimed at investigating whether human testicular germ cells (TGCs) can support such viral invasion by studying HIV interactions with TGCs *in vitro*. Our results indicate that isolated primary TGCs express alternative HIV-1 receptors, allowing virion binding but not entry. However, HIV-1 entered and integrated into TGCs upon cell-associated infection and produced low levels of viral proteins. *In vivo*, HIV-1 and SIV DNA was detected in a few TGCs. Molecular landscape analysis showed that TGCs have overall weak antiviral defenses. Altogether, our results indicate that human TGCs can support HIV-1 early replication, including integration, suggesting potential for endogenization in future generations.

## INTRODUCTION

Retroviruses have repeatedly infected the germ line during our evolution and integrated their DNA into the host genome, which has been passed on to the next generations by Mendelian inheritance (1, 2). When not detrimental, the integration of viral sequences has led to their fixation and endogenization in the population (3). Thus, about 8% of the human genome is now composed of endogenous retroviruses (ERVs), representing 31 distinct viral groups (2). Such integration, still ongoing in mammals (4), has driven the acquisition of new functions in the host, including a protective role against exogenous infections (5, 6). Within the retrovirus family, several lentiviruses have been endogenized in mammals, including simian immunodeficiency virus (SIV) in pro-lemurians (7–12). A few nonretroviruses have also colonized the germ line of their host, but the mechanisms for their integration are unclear (1).

Human immunodeficiency virus type 1 (HIV-1) is an emerging zoonotic lentivirus that has infected over 70 million people since its worldwide spreading in the human population from the beginning of the 1980s. Current antiretroviral treatments effectively control but do not eradicate the virus, which still represents a major threat for the human population, with an average of nearly 2 million new infections each year. Horizontal transmission through semen plays a key role in HIV-1 dissemination and is most likely mediated by the free viral particles and infected leukocytes present in this fluid (13). We and others found that HIV/SIV strains in semen are locally produced in a subset of individuals (reviewed in reference 13) and arise from several organs within the male genital tract (14). Interestingly, HIV-1 can associate with spermatozoa *in vitro* (15) and was detected by some authors in a small proportion of patients’ sperm (16–25). Since spermatozoa have a highly compact nucleus and are transcriptionally silent, this detection may suggest infection of their germ cell progenitors in the testis.

The testis is an immune-privileged organ with restricted drug penetration, considered to constitute a tissue reservoir for HIV and other emerging viruses such as Zika and Ebola (1, 26, 27). HIV-1 productively infects testicular T lymphocytes and resident macrophages, which are naturally localized in close proximity to the early germ cells present in the basal compartment of the seminiferous epithelium and therefore outside the blood-testis/Sertoli cell barrier (27–31). A couple of early studies reported HIV nucleic acids in isolated cells resembling germ cells within the seminiferous tubules of deceased patients performed with *in situ* PCR, a controversial technique suspected of generating false positives (30, 32, 33). These findings have therefore been largely dismissed (34, 35). Furthermore, the detection of viral RNA (vRNA) in TGCs could reflect an accumulation of virions bound to the cell surface rather than a true infection. Unfortunately, scarce access to the testicles of HIV-1^+^ men has hindered further investigations on this debated issue (35). SIV RNA and proteins were later described in TGCs from nonhuman primates (NHPs), using *in situ* hybridization and immunohistochemistry (36, 37). Whether human TGCs are infected by HIV and to what extent remain an open question.

In this study, we aimed to investigate whether human TGCs support HIV entry and integration as a proxy to evaluate the potential for viral colonization in the next generations of the human genome. We found that HIV-1 binds to primary TGCs, but that viral entry was inefficient. However, virus integration and early viral protein expression were observed following cell-associated infection. *In vivo*, testicular germ cells harboring viral DNA were detected in the testis from an HIV-infected individual by the next-generation *in situ* hybridization DNAscope. SIV-infected TGCs were also detected in one experimentally infected rhesus macaque and one African green monkey, a natural host for SIV. To determine whether human TGCs had evolved elevated defense mechanisms to prevent vertical transmission of viral sequences to the offspring and thus potential endogenization, we analyzed the molecular landscape of TGCs and compared it with that of HIV permissive and nonpermissive testicular somatic cells by using single-cell RNA-sequencing data. This analysis revealed relatively low gene expression levels for viral sensors and HIV early life cycle inhibitors in TGCs, together with an enrichment in HIV early cofactors in spermatogonia. Overall, our study provides the proof of concept that human TGCs can support HIV entry and integration, albeit inefficiently. Colonization of the human male germ line could therefore lead to the vertical transmission of viral genes and ultimately to their endogenization in the next generations.

## RESULTS

### Isolation and characterization of primary human testicular germ cells.

**FIG 1:**
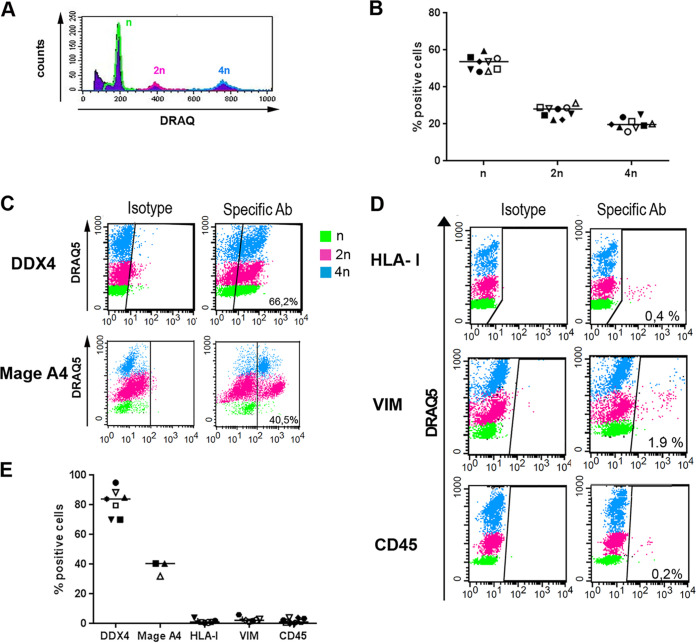
Characterization and purity of primary TGCs. (A and B) DRAQ5 DNA intercalating agent detection in flow cytometry on isolated TGCs showed the presence of haploid (n DNA [green]), diploid (2n DNA [pink]), and tetraploid (4n DNA [blue]) cells (representative profile in panel A). (C and E) As expected, DDX4 labeled most germ cells irrespective of their ploidy, whereas MAGEA4 labeled early diploid and tetraploid germ cells (representative profile in panel C). (D and E) Somatic cell contaminant analysis in isolated TGC populations labeled with DRAQ5 using flow cytometry and antibodies specific for somatic cells (class I HLA and vimentin) and leukocyte antigen CD45 (representative profile in panel D).

We isolated fresh TGCs from the testes of uninfected donors displaying normal spermatogenesis and characterized them based on their ploidy profile (Fig. 1A and B) as well as expression of specific markers (Fig. 1C to E). As expected, three DNA content profiles were present in all TGC preparations (*n* = 9 donors), with a median value of 54% of n DNA spermatids (range, 48 to 59%), 28% of 2n DNA spermatogonia and secondary spermatocytes (range, 22 to 31%), and 20% of 4n DNA primary spermatocytes and mitotic spermatogonia (range, 16 to 25%) (Fig. 1A and B). The germ cell marker DDX4 was detected in a median of 84% of TGCs (range, 70 to 95%) (Fig. 1C and E). As expected, DDX4 did not label every germ cell because its expression is variable among germ cells, independently of their differentiation stage (38). TGCs with n, 2n, and 4n DNA were DDX4^+^, with median values of 80% (72 to 98%), 61% (40 to 95%), and 90% (68 to 97%), respectively (Fig. 1C). The early germ cell marker MAGEA4, which labels undifferentiated 2n DNA spermatogonia up to 4n DNA pre-leptotene primary spermatocyte (39), was detected in 40% (32 to 40%) of TGCs (*n* = 3 donors), among which are 37% (24 to 41%) of 2n DNA and 76% (72 to 86%) of 4n DNA cells (Fig. 1C and E). The purity of TGC preparations was systematically >94%, as determined using somatic cell markers HLA class I (median, 1%; range, 0.4 to 4%) and vimentin (median, 2%; range, 1 to 6%), as well as the pan-leukocyte marker CD45 (median, 1%; range, 0 to 4%) (Fig. 1D and E).

### HIV binds to TGCs that express alternative receptors on their surface.

**FIG 2:**
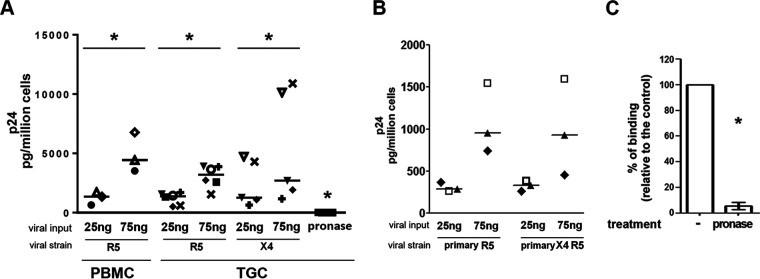
HIV-1 binding to primary TGCs. TGCs isolated from 9 donors or PBMCs used as a positive control were tested for their ability to capture (A) lab-adapted HIV-1 R5_SF162_ or HIV-1 X4_IIIb_ or (B) primary R5 and R5X4 strains following 2 h of incubation at 37°C with the indicated amount of virus, as measured by p24 ELISA on cell lysates following thorough washes and deduction of p24 background measured in wells with no cells. Pronase treatment postbinding was used as a negative control to ensure measure specificity. Each donor is represented by a specific symbol. (C) Pronase treatment of TGCs prior to HIV R5_SF162_ exposure (25 ng p24) abrogated viral binding (*n* = 5). Statistical analysis was performed with the nonparametric Wilcoxon test. *, *P* < 0.05.

We first determined whether HIV can attach to TGCs by using the well-characterized R5 macrophage-tropic HIV-1_SF162_ and X4 HIV-1_IIIB_ strains (Fig. 2). Dose-dependent binding to TGCs was observed for both strains (Fig. 2A). Pronase treatment effectively removed bound HIV-1, indicating that the majority of the attached virions remained at the cell surface (Fig. 2A). Similar findings were observed for a primary R5 non-macrophage-tropic isolate and a primary R5X4 HIV-1 strain (Fig. 2B). Incubation of TGCs with a protease prior to viral exposure decreased HIV-1 p24 detection in cell lysates by a median of 97% (range, 81 to 100%), indicating that cellular proteins mediated HIV-1 attachment to TGCs (Fig. 2C).

**FIG 3:**
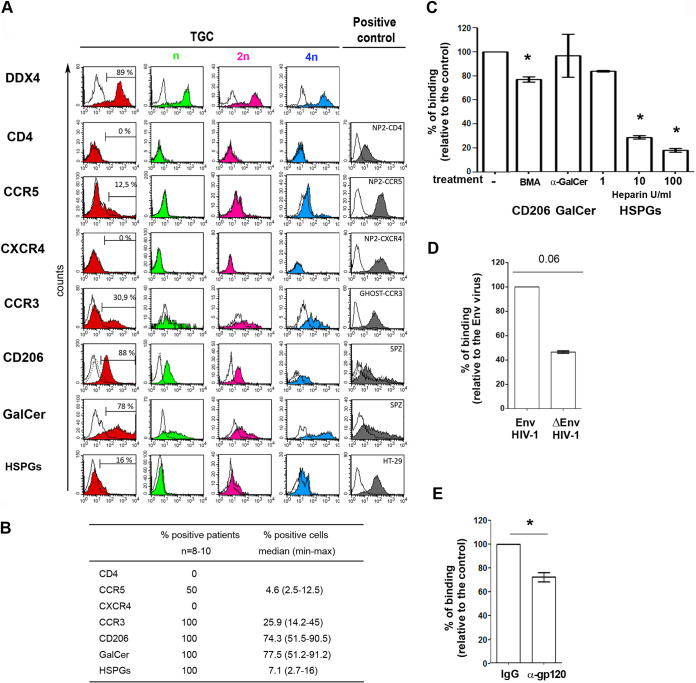
HIV-1 alternative receptor expression by primary TGCs and HIV-1 binding inhibition. (A) Representative profiles of membrane expression of HIV receptors on the whole population of TGCs (red) and the n (green), 2n (pink), and 4n DNA (blue) TGC populations. Immunolabeling was performed using specific antibodies against CD4, CCR5, CXCR4, CCR3, GalCer, or HSPGs or, in the case of CD206, after detection of biotinylated BSA-mannose (BMA) ligand in the presence (dot-lined histograms) or absence (filled histograms) of mannan competitor. BSA was used as a control (open histograms). Cells were further stained with DRAQ5 for ploidy profile. Specific antibody detection was ensured using isotypes as controls, except for HSPGs, where pronase treatment was used. The right panel shows positive controls for each receptor. (B) The table shows for each receptor the percentage of positive patients and the median and range of percentages of positive cells. (C) HIV binding was measured after incubation of TGCs with HIV R5_SF162_ (25 ng p24) in the presence or absence of CD206 BSA-mannose competitor (BMA), galactosylceramide-specific monoclonal antibody, or HSPG competitor (heparin) at the 3 indicated doses. Results represent the mean ± standard error of the mean (SEM) from 3 to 6 independent experiments performed in duplicate and are expressed relative to their respective controls (virus-cell incubation without receptor inhibitors or in the presence of BSA for BSA-mannose). (D) TGCs obtained from 4 donors were incubated for 2 h at 37°C with the *env* gene deleted or HIV-1 env-expressing pseudoviruses (25 ng p24), and p24 antigen was measured by ELISA. (E) Viral env neutralization was performed in the presence of gp120-specific antibody (*n* = 3). In all experiments, background p24 levels measured in wells without cells were deduced from the values obtained with cells. Statistical analysis was performed with the nonparametric Wilcoxon test. *, *P* < 0.05.

We next explored the expression of candidate receptors for HIV on the cell surface of TGCs (Fig. 3A and B). In agreement with our previous immunohistochemistry data (27), TGCs did not express the main HIV receptor CD4 (Fig. 3A and B). The chemokine HIV coreceptor CCR5 was detected in 4 out of 8 donors on the surface of a very low proportion of TGCs (median, 5%; range, 2 to 12%), whereas CXCR4 was absent in all donors. The chemokine receptor CCR3 was expressed at the TGC surface in all donors, with a median of 26% (14 to 45%) positive cells (Fig. 3A and B). The alternative HIV binding molecules CD206/mannose receptor, galactosylceramide (GalCer), and heparan sulfate proteoglycans (HSPGs) were detected at the surface of TGCs from all donors, with medians of, respectively, 74% (51 to 90%), 77% (51 to 91%), and 7.1% (3 to 16%) positive cells (Fig. 3A and B). These receptors were expressed by TGCs irrespective of their DNA content (see representative profiles in Fig. 3A). Competition experiments with bovine serum albumin (BSA)-mannose (BMA), a ligand for CD206, induced a median reduction of virus attachment of 24% (range, 16 to 29.1%) (Fig. 3C). In contrast, neutralizing antibodies against the cellular glycolipid galactosylceramide had no effect on HIV attachment to TGCs (Fig. 3C), in agreement with the protease experiments. Heparin at 100 U/ml strongly inhibited the capture of HIV particles (median inhibition of 82%; range, 79 to 86%) (Fig. 3C). The contribution of the viral envelope (env) protein gp120 to binding was assessed using pseudoviruses with the *env* gene deleted and antibodies against gp120 (Fig. 3D and E). In 4 independent experiments, the capture of HIV-1 by TGCs was reduced by a median of 53.4% (range, 52.6 to 54.7%) in the absence of *env* (Fig. 3D), while gp120 neutralizing antibodies reduced HIV binding to TGCs by a median of 29% (range, 17 to 39%) (Fig. 3E), indicating that specific interactions of gp120 with cell surface receptors favored attachment.

### TGCs support HIV-1 entry and integration.

**FIG 4:**
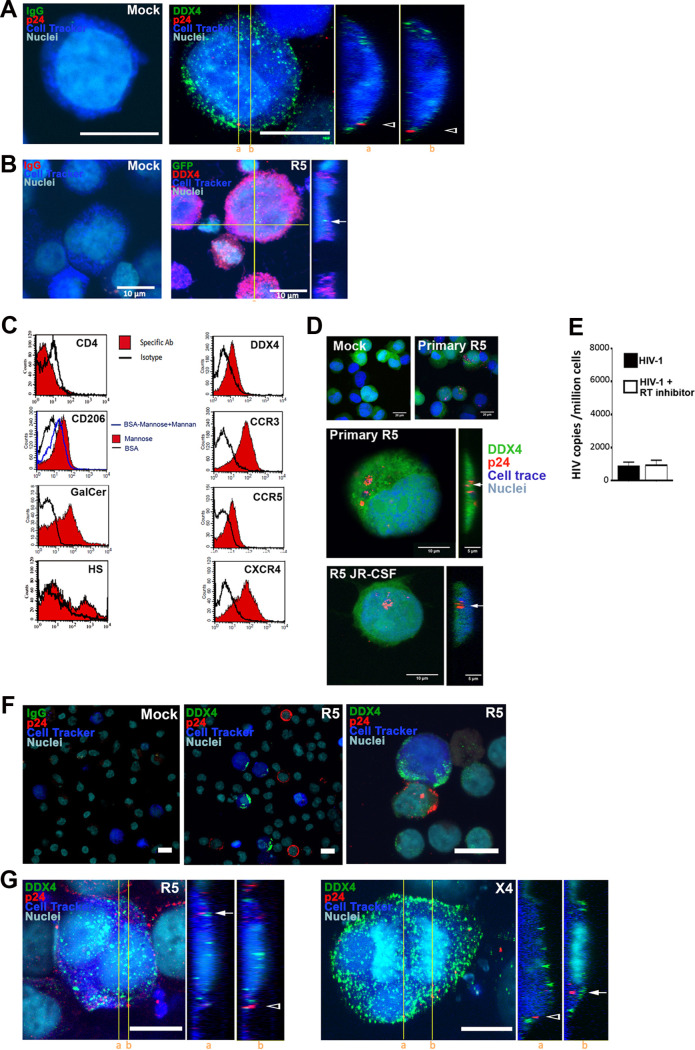
HIV entry in primary TGCs following cell-associated infection. (A) TGCs prelabeled with CellTracker (blue) were exposed for18 h to cell-free wild-type R5 HIV-1 or mock exposed. Cells were costained for p24 (red) and DDX4 (green), and p24 localization was assessed by confocal microscopy “Mock” shows absence of labeling with DDX4 antibody isotype (IgG). (B) TGCs prelabeled with CellTracker (blue) were exposed for 18 h to R5 HIV-1^GFP-Vpr^ viral particles (green) or mock exposed. Rare viral entry events (arrows) in cells labeled with DDX4-specific antibody (red) were visualized by confocal microscopy. “Mock” shows absence of labeling with DDX4 antibody isotype (IgG). (C) Potential HIV receptors were assessed on the surface of Tcam-2 cells labeled for DDX4 using specific antibodies against CD4, CCR5, CXCR4, CCR3, galactosylceramide (GalCer), and HSPGs or following detection of BSA-mannose, a ligand for mannose receptor. Immunolabeling was analyzed by flow cytometry. (D) HIV-1 entry (arrows) was assessed in CFSE-labeled Tcam-2 cells (in green), exposed for 4 h to either a primary R5-tropic HIV-1 strain (ES X-2556-3) or HIV R5 JRCSF or mock exposed, before immunolabeling with p24 antibody (red). Nuclei were stained with DAPI (blue). (E) HIV-1 reverse transcription was assessed by quantitative PCR (qPCR) on Tcam-2 cells cultured for 24 h with or without the reverse transcriptase inhibitor nevirapine, following exposure to HIV R5_JR-CSF_ (*n* = 6). (F and G) TGCs prelabeled with CellTracker (blue) were incubated for 18 h with either PBMCs infected with a primary R5-tropic HIV-1 strain or mock infected (F) or with Jurkat cells infected with HIV-1 R5 or X4 strains (G). Cells were costained for p24 (red) and DDX4 (green) or with control IgG (shown here as “Mock”). (A, B, and G) z-stack maximum projections are presented along with cross-sectional yz orthogonal viewing from the indicated positions (a and b) to discriminate entering viral particles (white arrows) from membrane-bound viruses (arrowheads). Nuclei are stained with DRAQ5 (cyan). Scale bars = 10 μm.

We next investigated the ability of HIV to enter TGCs and process postentry steps of viral replication. A major challenge for the study of primary TGCs *in vitro* is their low survival rate outside the testis environment, since they require physical contacts and paracrine exchange with feeder somatic cells for their maintenance and development. To overcome this challenge, we set up a coculture of primary TGCs with human testicular somatic cells in a specific medium that allows long-term maintenance of early germ cells (26). In this culture system, viability dyes showed over 80% of live TGCs after 12 days. After incubation of freshly isolated TGCs with wild-type HIV-1 R5 (SF162, BaL, and JR-CSF) and X4 (IIIB) strains for 4 h or overnight, HIV p24 was detected on the cell surface but not within the cells (Fig. 4A). Using a fluorescent HIV-1^GFP-Vpr^ isolate, only rare entry events were visualized (Fig. 4B). To further dissect HIV interactions with testicular germ cells, we also analyzed the well-characterized human testicular germ cell line T-cam2, which displays characteristics of stem spermatogonia (40, 41), for HIV receptor expression and replication. Except for CXCR4, T-cam2 cells displayed similar HIV receptor expression to primary TGCs (Fig. 4C), and HIV cell-free entry was also inefficient (Fig. 4D and E). Because cell-associated infection is much more efficient than cell-free infection, and within the testis early TGCs are close to interstitial lymphocytes and macrophages, we investigated HIV entry and integration in TGCs incubated overnight with HIV-1-infected lymphocytes. Confocal microscopy showed DDX4-positive TGCs next to HIV-1-infected peripheral blood lymphocytes (PBLs) or Jurkat T cells (Fig. 4F) and revealed viral uptake of HIV-1 p24 by DDX4^+^ TGCs, some in division (Fig. 4G). Altogether, these results indicate that HIV-1 can enter primary TGCs in a cell-associated manner, whereas in our experimental model, cell-free infection was not readily detected.

**FIG 5:**
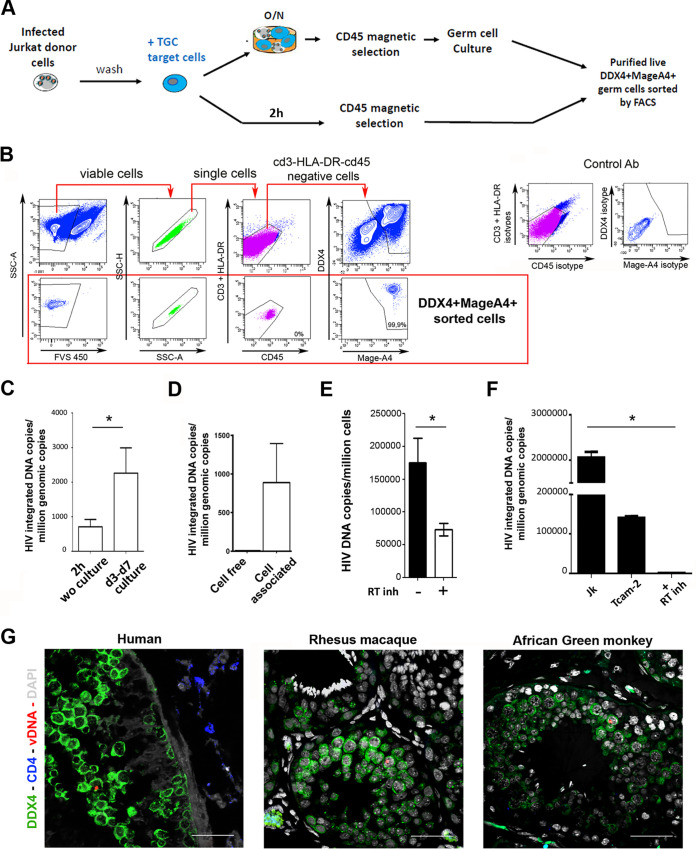
HIV-1 integration in testicular germ cell genome and *in vivo* detection of HIV and SIV DNA in human and nonhuman primate testicular germ cells.(A to C) HIV-1 integration in primary TGCs. (A) Experimental design. After overnight contact with Jurkat cells infected with HIV-1 *nef*-IRES-GFP, TGCs were recovered following CD45 magnetic selection and placed in culture for 5 days. The cells were then submitted to FACS sorting for live cells, cells negative for leukocyte markers CD45, CD3, and HLA-DR, and cells positive for germ cell markers DDX4 and MAGEA4. As a negative control, TGCs purified by CD45 magnetic selection immediately after contact with Jurkat cells were sorted by FACS similarly to the samples incubated overnight. (B) Gating strategy and representative profile of purified live germ cells. Live single cells were selected based on absence of detection of leukocyte markers CD3, HLA-DR, and CD45 and on positive expression of the DDX4 and MAGEA4^+^ germ cell markers. Control antibodies are shown in the bottom panel. (C) HIV-1 integrated DNA was measured by Alu-Gag PCR of 10,000 sorted TGCs exposed to Jurkat cells for 2 h without culture or after 3 to 7 days of culture following overnight exposure to Jurkat cells, in 3 independent experiments. Statistical analysis was performed with the nonparametric Wilcoxon test. *, *P* < 0.05. (D to F) HIV integration in Tcam-2 cells. (D) HIV-1 integrated DNA was measured on Tcam-2 cells either cultured for 48 h following exposure to R5_JR-CSF_ HIV-1 (cell free) or exposed overnight to Jurkat cells infected with HIV R5_JR-CSF_, purified by CD45 magnetic selection, cultured for 5 days, and FACS sorted for CD45-negative live cells (cell associated). (E and F) HIV-1 reverse transcription and integration were assessed on Tcam-2 cells exposed to VSV-G-pseudotyped HIV-1. (E) Reverse transcripts were detected after 24 h of culture with or without the reverse transcriptase inhibitor nevirapine (*n* = 6), and (F) HIV-1 integration was assessed by Alu-Gag PCR in Jurkat cells (Jk) and in Tcam-2 cells in the presence or absence of reverse transcriptase inhibitor (*n* = 3). (G) *In vivo* detection of HIV and SIV DNA in human and nonhuman primate testicular germ cells by DNAscope. Shown are representative images of TGCs (green) harboring viral DNA (red) within testis tissue sections from one HIV-1-infected man (viral load [VL] = 51,681 copies [cp]/ml), one chronically infected rhesus macaque (VL > 10^6^ cp/ml), and one African green monkey 64 days postinfection (VL = 12,085 cp/ml). Scale bars = 100 μm.

To determine whether HIV DNA could integrate into the germ cell genome *in vitro*, freshly isolated TGCs were exposed to infected Jurkat T cells overnight, which were subsequently removed using anti-CD45 magnetic beads. TGCs were cultured for 3 to 7 days, and viable cells were sorted by flow cytometry, based on DDX4 and MAGEA4 expression and absence of leukocytes and somatic cell markers CD45 and CD3, as well as HLA-DR detection (Fig. 5A and B). As a control, TGCs were mixed with infected Jurkat donor cells (2 h of contact) and processed without further incubation and culture, following the same protocol (magnetic beads and fluorescence-activated cell sorter [FACS] sorting) as TGCs incubated overnight with donor cells (Fig. 5A). Following the cell selection procedure of cultured TGCs, most live germ cells were double positive for DDX4 and MAGEA4 (median, 92%; range, 82.6 to 99%), indicating TGCs in the premeiotic up to early meiotic stage. In 3 independent experiments, sorted cultured germ cells were >99% positive for DDX4 and MAGEA4 (median, 99.2; range, 99 to 99.9%) and negative for immune cell markers CD45, CD3, and HLA-DR (representative profile in Fig. 5B). Alu-Gag PCR performed on isolated TGCs demonstrated the presence of integrated HIV DNA in sorted cultured early germ cells from 3 out of 3 donors (Fig. 5C). Exposure of T-cam2 cells to Jurkat T cells infected with HIV-1 R5 and X4 strains also led to the detection of integrated HIV-1 DNA after flow cytometry sorting of live cells negative for CD45 in 3 independent experiments, whereas no integrated viral DNA was detected after cell-free infection (Fig. 5D). To bypass cell-free HIV entry restrictions, T-cam2 cells were infected with HIV-1 pseudotyped with vesicular stomatitis virus (VSV) envelope (VSV-G). HIV DNA became readily measurable in T-cam2 cells, and its level was significantly decreased by the reverse transcriptase (RT) inhibitor nevirapine, indicating active reverse transcription of the viral genome (Fig. 5E). Integrated HIV-1 DNA was detected in T-cam2 cells’ genome exposed to cell-free HIV pseudotyped with VSV-G using Alu-Gag PCR (Fig. 5F). This detection was abrogated in the presence of nevirapine (Fig. 5F).

Altogether, these results indicate that isolated early germ cells can support a low level of HIV-1 DNA integration into their genome.

### HIV-1/SIV DNA is present in TGCs *in vivo*.

We next aimed to assess whether TGCs harboring HIV DNA are present *in vivo*. As mentioned, samples of testis tissue from HIV-infected men are extremely rare. Nevertheless, we managed to have access to the testis of one deceased HIV^+^ patient. We screened for HIV DNA^+^ TGCs using next-generation DNAscope *in situ* hybridization for HIV DNA combined with immunofluorescence detection of germ cell marker DDX4. Isolated DDX4^+^ germ cells harboring viral DNA were found within morphologically normal seminiferous tubules (Fig. 5G). To investigate whether human TGCs are more restrictive to HIV infection than TGCs from nonhuman primates to SIV, we performed the same approach on the testis from one rhesus macaque and one African green monkey experimentally infected with SIV. In both the rhesus macaque and the African green monkey, isolated HIV DNA^+^ TGCs were also observed (Fig. 5G). Overall, these results indicate that human TGCs can support a low level of HIV replication up to DNA synthesis *in vivo*, similarly to their nonhuman primate counterparts, which could represent a way for HIV to become endogenous in the offspring.

### TGCs support a low level of HIV replication.

**FIG 6:**
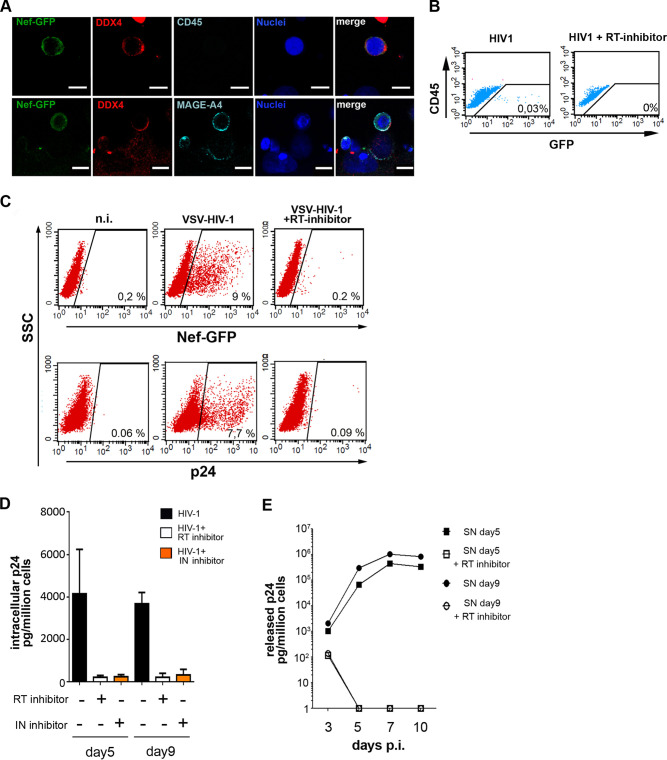
HIV replication in TGCs. (A and B) Early HIV-1 replication in germ cells following contact with Jurkat cells infected with HIV-1 *nef*-IRES-GFP. (A) TGCs incubated with infected Jurkat cells were purified using magnetic bead selection for CD45 and cultured for 9 to 12 days before colabeling for DDX4 and CD45 (A, upper panel) or DDX4 and MAGEA4 markers (A, lower panel). Confocal images show DDX4^+^ germ cells and MAGEA^+^ DDX4^+^ early germ cells expressing Nef-GFP. Nuclei were stained with DAPI (blue). Scale bars = 10 μm. (B) Tcam-2 cells incubated for 18 h with infected Jurkat cells and further cultured for 9 days with or without nevirapine. Cells were stained with CD45-PE to discriminate Tcam-2 cells (CD45 negative) from residual Jurkat cells (CD45 positive) and analyzed by flow cytometry for Nef-GFP expression. The numbers of GFP-positive events are indicated in the plot. (C to E) HIV-1 replication after Tcam-2 exposure to VSV-G-pseudotyped HIV-1. Expression of viral protein Nef-GFP and p24 was detected by flow cytometry in Tcam-2 cells cultured for 5 days with or without nevirapine (C). Viral production in infected T-cam2 cells cultured for 5 days and 9 days with or without RT or integrase inhibitor (raltegravir) was assessed by measuring intracellular p24 in ELISA (D). Infectious viral particle release was assessed by exposing PBMCs for 4 h to T-cam2 cell supernatants collected at days 5 and 9 postinfection. p24 was measured by ELISA in supernatants from exposed PBMCs at the indicated time points (E). The data shown are representative of 3 independent experiments.

Having shown that TGCs can harbor HIV DNA, we aimed to determine whether these cells could support HIV protein expression and virion production. Viral protein expression in TGCs was analyzed following overnight exposure to Jurkat T cells infected with HIV-1 bearing a *nef*-internal ribosome entry site (IRES)-green fluorescent protein (GFP) construct. Nef-GFP was detected using confocal microscopy in primary DDX4^+^ and MAGEA4^+^ TGCs after 9 to 12 days of culture (Fig. 6A). Similarly, a low level of Nef-GFP was detected by flow cytometry in CD45-negative T-cam2 cells after cell-associated infection, and this detection was abolished by pretreatment of T-cam2 cells with the RT inhibitor nevirapine (Fig. 6B). Following exposure of T-cam2 cells to VSV-G-pseudotyped HIV-1-bearing *nef*-IRES-GFP, up to 9% of germ cells expressed Nef-GFP at day 5 postinfection, whereas less than 0.2% of the cells were positive in the presence of nevirapine (Fig. 6C). Inhibitors of both HIV reverse transcriptase (nevirapine) and integrase (raltegravir) similarly impaired the expression of intracellular p24 (Fig. 6D), demonstrating expression of viral proteins from *de novo* reverse-transcribed and integrated virus. The release of infectious particles by T-cam2 cells was confirmed by the infection of permissive human PBLs with T-cam2 supernatants collected at days 5 and 9 postinfection (Fig. 6E). Altogether, these results show that in addition to integration, early TGCs can support a low level of virus production.

### Spermatogonia are enriched in HIV early cofactors compared with testicular somatic cells.

**FIG 7:**
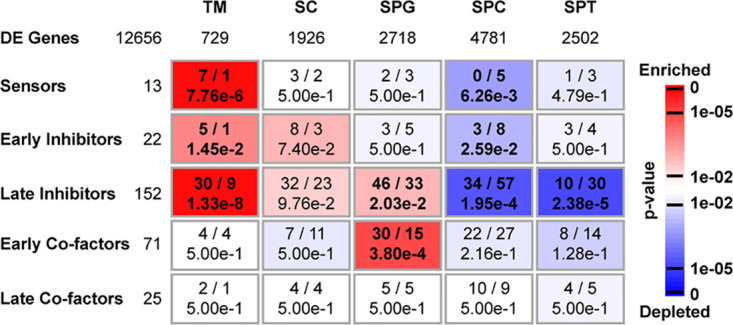
Enrichment analysis of factors involved in the HIV life cycle in the human testis. Previously published single-cell RNA-sequencing data for human adult testis (85) were used to identify differentially expressed (DE) genes showing peak expression in testicular macrophages (TMs; 729 genes), Sertoli cells (SCs; 1,926 genes), spermatogonia (SPGs; 2,718 genes), spermatocytes (SPCs; 4,781 genes), or spermatids (SPTs; 2,502 genes), including 283 factors involved in the HIV life cycle (out of an initial set of 335 factors retrieved from the literature). The over- or under representation of these factors considered according to different categories (“Early Co-factors,” “Late Co-factors,” “Sensors,” “Early Inhibitors,” and “Late Inhibitors”) was then evaluated in each expression cluster by means of a hypergeometric test. Rectangles indicate the observed (left) and expected (right) numbers of genes for each type of factors in each expression cluster, as well as the corresponding *P* values adjusted by the Benjamini-Hochberg procedure. Enrichments and depletions are indicated in red and blue, respectively, according to the scale bar.

To further evaluate the permissiveness of TGCs to HIV replication postentry, we compared the expression profiles of 335 factors that promote or inhibit the HIV-1 life cycle postentry among spermatogonia (SPG), spermatocytes (SPCs), and spermatids (SPTs) with that in two distinct testicular somatic cell types: testicular macrophages (TMs), which are permissive to HIV, and Sertoli cells (SCs), which are not a target for HIV, using single-cell RNA-sequencing data (42) (see Table S1 in the supplemental material). As expected for an immune cell type, viral sensors (*P* = 7.76e−6) and early inhibitors (*P* = 1.45e−2) were overrepresented in TMs. This is in contrast to TGCs, among which sensors and early inhibitors were even depleted in SPCs (*P* = 6.26e−3 and 2.59e−2, respectively), whereas SCs showed an intermediate profile between those of TGCs and TMs (Fig. 7). SPG were significantly enriched in late inhibitors (*P* = 2.03e−2), although less so than TMs (*P* = 1.33e−8), whereas SPCs (*P* = 1.95e−4) and SPTs (*P* = 2.38e−5) were depleted (Fig. 7). Interestingly, early cofactors spanning reverse transcription up to DNA integration steps were overrepresented in SPG (*P* = 3.80e−4) but not in TMs and SCs, and tended to be depleted in SPTs (albeit not significantly), suggesting a gradient of reduced permissiveness for HIV early replication steps as TGCs differentiate. The distribution frequencies of late cofactors (most of them implicated in viral transcription) were comparable for all cell types (Fig. 7). Altogether, these data indicate that TGCs have overall a lower level of innate sensing equipment and low proportion of early HIV inhibitors compared with SCs and TMs. Due to their specific enrichment in early cofactors, SPG may represent a more permissive environment for HIV postentry early steps up to viral DNA integration than late germ cells. Nevertheless, SPG are also enriched in late inhibitors, which could explain low virus production.

## DISCUSSION

Here, we revealed that primary testicular germ cells can support HIV-1 genome integration and early viral protein production upon contact with infected lymphocytes *in vitro*. In contrast, cell-free virus infection did not lead to detectable viral entry despite HIV-1 binding to TGCs and alternative receptor expression. DNAscope analysis of the testis of one infected human and nonhuman primates demonstrated the presence of isolated HIV or SIV DNA^+^ TGCs. Using the stem spermatogonia cell line T-cam2, we confirmed that viral integration occurred following cell-associated infection or after bypassing HIV-1 entry and showed that these early germ cells can produce a low level of infectious virions. Gene expression analysis of an array of factors affecting the viral life cycle showed that TGCs were not enriched in defense factors compared with testicular macrophages and Sertoli cells and that spermatogonia were specifically enriched in cofactors involved in the HIV postentry life cycle up to integration.

While a number of viruses use ubiquitous receptors to attach to and enter the cells, HIV virions require specific cellular receptors for infection. Although the main HIV receptor CD4 was absent, TGCs expressed on their surface CD206, galactosylceramide (GalCer), and HSPGs, all previously shown to allow binding, entry, or capture of a range of viruses, including HIV in various cell types (42–49). Ligands of HSPGs, and to a lesser extent of CD206, inhibited binding of cell-free virions to TGCs, whereas despite its broad expression, GalCer was not involved—possibly because the detected isoforms were unable to bind gp120 (50). HIV binding to TGC only partly involved HIV envelope gp120, which could be due to HIV envelope-independent uptake by HSPGs, as previously described in other cells (51). These findings are comparable to that in spermatozoa (15, 43–45) and indicate that these receptors for viruses are retained on the TGCs’ surface as they differentiate into spermatozoa. This implies that early TGCs located below the Sertoli cell tight junctions could act as a Trojan horse, enabling the virions attached to their surface to cross the blood-testis barrier as these cells progress to the seminal lumen during their differentiation. Bypassing the blood-testis barrier could allow the virus to shelter from immune recognition by antibodies and immune cells, favoring viral reservoir establishment in the testis. It could also facilitate the release of viral particles into semen and their attachment to sperm.

In our experimental system, HIV entry and reverse transcription were not detected in primary TGCs exposed to cell-free wild-type virus. In CD4-negative somatic cells, free viral particle entry has been reported *in vitro* but was overall inefficient, and viral internalization by vesicular uptake was essentially a dead end with respect to productive infection (46, 52–59). Despite free virus entry limitation, HIV-1 nucleic acids have been evidenced in HIV^+^ individuals *in vivo* in specific CD4-negative cell types such as renal epithelial cells (60). Although the mechanism for HIV entry into these CD4-negative cells *in vivo* remains elusive, studies have suggested it may rely on cell-associated infection (54, 59, 61–63). Indeed, cell-to-cell contact between infected and noninfected target cells mediates the transfer of HIV-1 to recipient cells with much greater efficiency (100 to 1,000 times) than direct exposure of target cells to cell-free virus (64) and led to viral replication in a few CD4-negative cells: e.g., renal epithelial cells and astrocytes (54, 59, 63). In the testis of humans and nonhuman primates, T lymphocytes and interstitial macrophages infected with HIV/SIV are in close proximity to spermatogonia (28, 30, 36, 37), which are located at the basal compartment of the seminiferous epithelium and therefore not segregated by the blood-Sertoli cell/testis barrier. Here, we showed that coculture of infected T cells and TGCs, as well as bypassing HIV entry steps with VSV envelope-pseudotyped HIV-1, led to HIV DNA integration into the testicular germ cell’s genome. Indeed, integrated viral DNA was measured in TGCs by Alu-Gag PCR and was inhibited by an RT inhibitor, demonstrating the specificity of this detection. In addition, early viral protein synthesis was observed in labeled germ cells by confocal microscopy, an indicator of viral integration since unintegrated HIV-1 DNA is associated with transcriptional silencing (65). Finally, viral protein detection in TGCs by flow cytometry was inhibited by both RT and integrase inhibitors.

Using next-generation *in situ* hybridization, we aimed to determine whether TGCs are infected by HIV *in vivo*. Our results demonstrate the presence of isolated DDX4^+^ testicular germ cells harboring HIV/SIV DNA in humans, as well as in an experimental simian model of HIV infection (rhesus macaque) and a natural host for SIV (African green monkey), indicating that the virus can proceed to early replication steps in both human and simian TGCs *in vivo*. However, HIV and SIV DNA expression in TGCs was low, which might reflect a requirement for cell-associated infection and low frequency of TGCs’ interactions with the infected leukocytes present in the interstitial tissue. Restriction factors could also be at play (see the next paragraph below). Due to the rarity of testis samples from infected individuals, we could not establish the frequency of HIV DNA detection in TGCs *in vivo*. However, our *in vitro* results on TGCs together with our analysis of the testis from an HIV^+^ donor suggest it is likely to be low. The detection of HIV/SIV within differentiating germ cells distant from the base of the seminiferous tubules *in situ* could potentially result from clonal infection of early TGCs. Importantly, our data indicate that early TGCs remained viable in culture after viral entry, integration, and protein expression, suggesting that viral integration was not detrimental to these cells. Clonal infection of the male germ line up to spermatozoa is suggested by reports of HIV DNA in a low percentage of ejaculated sperm (16–21, 23). Indeed, spermatozoa do not support HIV entry, are transcriptionally silent, and have a highly compact chromatin, thus preventing HIV replication steps. Patients’ spermatozoa harboring viral DNA *in vivo* were reported to fertilize hamster ova and transfer the HIV genome to early embryos (22), indicating that HIV did not impair their fertilization capacities.

The efficiency of HIV replication within a given cell type depends upon a fine balance between factors that positively (cofactors) or adversely (inhibitors) impact the virus life cycle, some of which are actively counteracted by HIV-1 proteins (e.g., APOBEC3G, BST2, and SERINC5/3) (66). Using single-cell transcriptomic data from human testis, we analyzed the expression levels of 335 transcripts that affect HIV replication postentry in isolated testicular germ cell populations and compared them with those in two somatic testicular cell types, an immune cell type infected by HIV *in vivo* (testicular macrophages) and a nonimmune cell type that is not infected by HIV (Sertoli cells) (27). Although we screened for a large number of known factors, we acknowledge that this analysis is not exhaustive and is restricted to transcripts. We found that TGCs were not enriched in viral sensors or early inhibitors. In spermatogonia, except for genes encoding the viral RNA (vRNA) sensor RIG-1 and its adaptor, IRF3, genes encoding viral RNA, DNA, or protein sensors either were not expressed (e.g., genes encoding Toll-like receptors [TLRs]), or their necessary cofactors were missing (e.g., IRF7, MyD88, and STING), indicating restricted sensing equipment. Transcripts for a range of early HIV inhibitors, including IFITM2 and -3 (fusion), PSG-L1 (reverse-transcription), OAS (uncoating), and Mx2 (nuclear import) were also reduced in TGCs versus Sertoli cells and/or testicular macrophages. However, TRIM28 was well expressed in TGCs compared with both Sertoli cells and testicular macrophages, which could explain limited HIV integration (Table S1). Interestingly, we recently demonstrated that human testicular germ cells were productively infected by Zika virus and that the infection did not impact their survival (26). In fact, testis antiviral responses were weak (26) and infected TGCs persisted in semen for a prolonged duration (91). We and others also showed that rodent germ cells exposed to a range of viral stimuli did not produce interferon-stimulated antiviral proteins (reviewed in reference 1). Altogether, these results suggest that aside from their naturally protective testis environment (e.g., blood-testis barrier and neighboring somatic cell types), testicular germ cells do not have strong canonical antiviral equipment. Further studies are needed to decipher how TGCs respond to viral infections and whether they have evolved specific alternative protective mechanisms to restrict viral replication and its potentially deleterious effects.

Interestingly, early HIV cofactors spanning from reverse transcription (e.g., DHX9 UBE2B and TRIAD3) to integration steps (LEDGF, INI1, and NUP153) were observed at higher frequency in spermatogonia compared with testicular macrophages, Sertoli cells, and late TGCs. We hypothesize that mitotic spermatogonia may therefore be more sensitive to HIV infection than late TGCs, all the more because their localization at the base of the seminiferous tubules could favor cell-to-cell infection by infected leukocytes, thus bypassing entry restriction. Because of their limited life span in culture, we could not determine whether spermatocytes and spermatids were less permissive to HIV replication than spermatogonia *in vitro*.

T-cam2 spermatogonia supported a low level of HIV-1 replication up to the production of infectious viral particles, as demonstrated by the infection of peripheral blood mononuclear cells (PBMCs) with infected T-cam2 supernatants and by the inhibition of T-cam2 virus release by RT and integrase inhibitors. *In vivo*, viral RNA and proteins have been detected in a few TGCs within the testis of SIV-infected macaques (36, 37), suggesting viral production in a restricted number of cells. Our transcriptomic data analysis revealed that spermatogonia are enriched in a wide range of viral transcription inhibitors (e.g., HDAC1 and -2, negative elongation factor SPT5, and NELF-A/B/CD), which could restrict late viral replication and hence virion production. Altogether, our results support the consensus that male gametes are at best minor contributors to HIV horizontal transmission in comparison with productively infected leukocytes and free viral particles present in semen (35, 67, 68). Bearing in mind that a normal ejaculate contains millions of spermatozoa (WHO reference range of 39 to 928 million), the chances for an individual to transmit HIV DNA to the genome of the offspring through oocyte fertilization with an HIV-infected sperm are naturally very low.

In conclusion, our study reveals that testicular early germ cells have the potential to support a low level of HIV integration upon cell-associated infection. Such infection could represent a way for HIV to integrate the germ line and become endogenous in the future, as has happened during human evolution for a number of retroviruses. Further studies are needed to determine the probability of this event at a population level.

## MATERIALS AND METHODS

### Primary testicular germ cell isolation and culture.

Normal testes were obtained at autopsy or after orchidectomy from prostate cancer patients who had not received any hormone therapy. The procedure was approved by the National Agency for Biomedical Research (authorization no. PF S09-015) and the CPP Ouest V local ethics committee (authorization no. DC-2016-2783). Only testes displaying normal spermatogenesis, as assessed by transillumination, were used in this study. For receptor detection and HIV binding assay, testes were dissociated with tweezers, incubated in digestion medium (0.5 mg/ml collagenase I, 75 μg/ml DNase, 1 μg/ml soybean trypsin inhibitor [SBTI], 1× phosphate-buffered saline [PBS]) for 90 min at 34°C under agitation (110 rpm) and then filtered (100-μm pore). Seminiferous tubules were then mechanically dissociated to avoid trypsin usage, before cell filtration through 300- and 100-μm-pore meshes in PBS containing DNase (40 μg/ml). For testicular cell culture and entry assays, testicular tissue fragments were first incubated in digestion medium (2 mg/ml hyaluronidase, 2 mg/ml collagenase I, 20 μg/ml in Dulbecco’s modified Eagle’s medium–Ham’s F-12 [DMEM/F-12]) for 60 min at 37°C under agitation (110 rpm). After centrifugation, the cell pellet was submitted to trypsin digestion (0.25%, 5 ml/g, for 20 min at 37°C) before trypsin inactivation with fetal calf serum (FCS), then filtered (60-μm pore), washed 3 times, and cultured overnight in DMEM/F-12 supplemented with 1× nonessential amino acids, 1× ITS (human insulin, human transferrin, and sodium selenite), 100 U/ml penicillin, 100 μg/ml streptomycin, and 10% FCS (all reagents from Sigma-Aldrich). Floating primary testicular germ cells were collected and cultured onto laminin (Sigma-Aldrich)-treated dishes at a density of 20,000 to 40,000 cells/cm^2^ in supplemented StemPro-34 (Invitrogen) as described elsewhere (69).

### Other cells and testis tissues.

Cells of the Tcam-2 seminoma cell line (70) (kindly given by Janet Shipley, The Institute of Cancer Research, London, United Kingdom) and CCR5-expressing Jurkat line (71) (provided by C. Goujon) were cultured in RPMI 1640 medium. Peripheral blood mononuclear cells (PBMCs) were prepared and cultured as previously described (72). 293T and positive-control NP2-CD4, NP2-CCR5, NP2-CXCR4, GHOST-CCR3, and HT-29 cells were maintained in Dulbecco’s modified Eagle’s medium (DMEM) supplemented with 10% fetal calf serum (FCS). All media were supplemented with 100 U/ml penicillin, 100 μg/ml streptomycin, 2 mM l-glutamine, and 10% FCS (all reagents from Sigma-Aldrich). Spermatozoa were obtained from healthy fertile volunteer donors with their informed consent (CPP Ouest V local ethics committee authorization no. DC-2016-2783) using a PureSperm gradient as instructed by the manufacturer (Nidacon Laboratories AB, Gotheburg, Sweden). Human testicular tissue was obtained at autopsy from an HIV-1-infected man (donor 108, with a blood viral load at death of 51,680 copies/ml) (73). Nonhuman primate (NHP) testicular tissues were collected at autopsy from an SIVagm^+^ African green monkey (*Chlorocebus sabaeus*) euthanized at 64 days postinfection for another study (blood viral load of 12,085 copies/ml) (74) and from a SIVmac251^+^ rhesus macaque euthanized at 100 days postinfection (blood viral load of >10^6^ copies/ml) (75).

### Viruses and DNA constructs.

Wild-type HIV-1 R5 macrophage-tropic (SF162, JR-CSF, and BA-L) or X4 (IIIb) strains and R5 non-macrophage-tropic (ES X-2556-3, subtype B) or R5X4 (ESP-2196-2, subtype G) primary isolates were obtained from the NIBSC (National Institute for Biological Standards and Control Centralised Facility for AIDS Reagents), and viral stocks were produced on PBMCs as previously described (72). The following viruses were generated by transiently transfecting 293T cells with Lipofectamine 2000 (Invitrogen): (i) *env*-deleted or HIV-1 env-expressing pseudoviruses using transfer vector pHRsin-cppt-SEW (76), packaging pCMV8.2 (77), and HxB2 env pSVIII (78) (kindly provided by Stuart Neil, King’s College, London, United Kingdom); (ii) HIV-1^GFP-Vpr^ viral particles, using pSG3Δenv, pcDNA3.1D/V5-His-TOPO-env_SF162_, GFP-Vpr (79) (a kind gift from Christiane Moog, University of Strasbourg, France); (iii) VSV-G-pseudotyped HIV-1, using full-length HIV-1 molecular clone pNL4-3 (80) or the R5-tropic pNL4-3 AD8 derivative (81) and VSV-G envelope-encoding plasmid (PT3343-5; Clontech); and (iv) VSV-G-pseudotyped HIV-1 *nef*-IRES-GFP, using pBR_NL4-3 IRES-eGFP (X4 tropic) or pBR_NL4-3 92TH014.12 IRES eGFP (R5 tropic) (82) (a kind gift from Frank Kirchhoff, Ulm University, Germany) and VSV-G envelope-encoding plasmid (PT3343-5; Clontech). 293T cell supernatants were collected 3 days posttransfection, filtered using 0.45-μm-pore membrane, and stored at –80°C.

### Flow cytometry for antigen detection and cell sorting.

Immunostaining of surface antigens was performed as previously described (83). The following antibodies were used: anti-CD45 (phycoerythrin [PE] or PC7 conjugated, HI30; BD Pharmingen), anti-HLA-ABC-PE (G46-2.6; BD Pharmingen), antivimentin (EPR3776, 1 μg/ml, Epitomics), anti-DDX4 (rabbit polyclonal, 5 μg/ml; Abcam), anti-MAGEA4 (clone 57B, 4 μg/ml) (39), anti-CD4 (ARP337, 10 μg/ml) and anti-CXCR4 (ARP3101 12G5, 10 μg/ml) (both from EVA/MRC, NIBSC), anti-CCR5 (45549, 10 μg/ml; R&D), anti-CCR3 (61828, 5 μg/ml; R&D), anti-galactosylceramide (mGalC, 10 μg/ml; Millipore), anti-heparan sulfate proteoglycans (10E4, 5 μg/ml; Seikagaku Corporation), and anti-p24 Gag RD1 (KC57; Beckman Coulter). PE-conjugated anti-mouse or anti-rabbit Igs (Jackson Immunoresearch) were used as secondary antibodies. Control isotype antibodies were used as negative controls, except for HSPGs, where cells were pretreated or not with pronase (3.5 mg/ml; Roche) and then subjected to immunostaining. The expression of mannose receptor (MR) was analyzed as previously described (15). Cellular DNA content was determined using DRAQ5 (20 μM; Biostatus Limited). A FACScalibur flow cytometer (Becton Dickinson, Franklin Lakes, NJ) and CELLQuestPro software were used for acquisition and analysis.

For cell sorting, TGCs were stained with the fixable viability stain FVS450 (BD Biosciences), as recommended by the supplier, before fixation in 1.5% paraformaldehyde. Cells were then labeled using DDX4 anti-rabbit PE-conjugated antibodies, CD45-PC7 antibody (Ab) (clone J33, 1/15; Beckman Coulter), CD3-BV510 Ab (clone HIT3a, 1 μg/ml; BD Biosciences), HLA-DR-BV510 Ab (clone G46-6, 7 μg/ml; BD Biosciences), and MAGEA4 Ab (clone 57B, 4 μg/ml) (39) previously coupled to Alexa Fluor 647 using the Zenon labeling kit (Molecular Probes). Matched isotype antibodies were used as negative controls. Cells negative for CD45, CD3, and HLA-DR and positive for DDX4 and/or MAGEA4 were sorted using a FACSAria flow cytometer (Becton Dickinson) connected to DIVA software. For Tcam-2, CD45-PC7 antibody was used for CD45-negative T-cam2 cell sorting.

### Binding assay.

The binding assay was performed as previously described (15), with some modifications. Briefly, cells were incubated with indicated HIV-1 strains (25 or 75 ng p24 per 10^6^ cells) in a final volume of 150 μl of medium and incubated for 2 h at 37°C in RPMI 1640 plus 2.5% FCS. Cells were then thoroughly washed and lysed, and the p24 content was determined by enzyme-linked immunosorbent assay (ELISA). When indicated, the assay was performed on cells exposed to pronase (3.5 mg/ml) or using virus preexposed to 1 to 100 U/ml heparin (Sigma-Aldrich). The role of mannose receptor was evaluated by incubating cells with HIV-1 in the presence of 100 μg/ml BSA-mannose (BMA; Sigma-Aldrich) or 100 μg/ml bovine serum albumin (BSA) as a control. Neutralization assays were performed by preincubating cells with antigalactosylceramide antibody (mGalC, 10 μg/ml for 30 min at 4°C; Millipore) or the viral inoculum with monoclonal antibody against HIV-1 gp120 (clone F105, 1 to 2 μg/ml for 30 min at 37°C; AIDS Reagents, NIBSC). Matched isotype antibodies were used as negative controls. Controls for nonspecific viral attachment were systematically performed by incubating the viral inoculum in the absence of cells. In all experiments, each condition was performed in duplicate and the residual p24 level was deduced.

### Cell infection.

TGCs and Tcam-2 and Jurkat cells were infected with 200 ng p24/10^6^ cells of the indicated virus as previously described (83) and cultured with or without 37.5 μM nevirapine (nonnucleosidic reverse transcriptase inhibitor) or 100 nM raltegravir (integrase inhibitor) (both from AIDS Reagents, NIBSC), as specified. For cell-associated infection, Jurkat donor cells were infected with the indicated VSV-G-pseudotyped HIV NL4-3 strain. TGCs or T-cam2 cells were incubated for 18 h with infected Jurkat cells (40 to 80% positive for HIV-1 Gag or GFP). Donor cells were then removed by positive selection of CD45^+^ cells (CD45 microbeads kit; Miltenyi Biotec) (TGCs) or pipetting (Tcam-2), and target cells were put into culture. GFP content was analyzed at the indicated time points in DDX4-positive cells by confocal microscopy (TGCs) or by flow cytometry in CD45-negative Tcam-2 cells. For integrated viral DNA quantification, TGCs and Tcam-2 cells were further purified by FACS sorting as described above (see “Flow cytometry for antigen detection and cell sorting”).

### Confocal microscopy for viral entry.

Germ cells were prelabeled with 10 μM carboxyfluorescein succinimidyl ester (CFSE) or CellTrace violet (Invitrogen) before being exposed to free viral particles overnight and treated with 0.25% trypsin-EDTA (Sigma-Aldrich) for 5 min at 37°C postinfection to remove virions attached to the cell surface. Cells were loaded onto poly-l-lysine-coated coverslips, fixed in 4% paraformaldehyde, washed in PBS, and incubated in a blocking/permeabilizing solution (0.3 M glycine, 0.05% Triton-X, 3% BSA, 1× PBS) for 30 min at room temperature. Cells were then stained with the following antibodies: mouse monoclonal anti-p24 (183-H12-5C; EVA, NIBSC), rabbit anti-DDX4 (5 μg/ml; Abcam), mouse anti-MAGEA4 (clone 57B, 4 μg/ml) (39), and CD45-Alexa Fluor 647 (J.33 clone; Beckman Coulter). Anti-mouse Alexa Fluor 555 (Thermo Fisher Scientific), anti-rabbit Alexa Fluor 488 (Thermo Fisher Scientific), and Alexa Fluor 647 (Jackson Immunoresearch) were used as secondary antibodies. Isotype control antibodies or mock-infected cells were used as controls. Nuclei were stained either with 10 μM DRAQ5 (Biostatus) or with DAPI (4′,6-diamidino-2-phenylindole; Molecular Probes), and slides were mounted with Prolong Gold antifade mountant (Molecular Probes). Images were acquired with the SP8 confocal system microscope (objective, 60×; Z-step, 0.3- to 0.5-μm intervals) (Leica) connected to LAS software and analyzed with Fiji software.

### Real-Time PCR for HIV-1 DNA.

Intracellular HIV-1 DNA was measured as previously described (73). Input virus was treated with Benzonase (500 U/ml; Sigma-Aldrich) before cell exposure to reduce the nucleic acid contamination in the viral stock. Cells infected in the presence of the RT inhibitor nevirapine (37.5 μM) were used to discriminate DNA that originated from input viruses. Integrated HIV DNA was quantified as previously described using an Alu-Gag PCR assay (84).

### Next-generation *in situ* hybridization (DNAscope).

DNAscope was used for the detection of viral DNA for both HIV and SIV, as previously described (75). To immunophenotype the cells containing vDNA, DNAscope detection by tyramide signal amplification (TSA) plus Cy3.5 was combined with immunofluorescence targeting cell markers by using rabbit polyclonal anti-DDX4 (1:500; Abcam) for TGCs and mouse monoclonal anti-CD4 (1:100, clone 1F6; Vector) for T-lineage cells. Sections of 4 to 6 5 μm were run per sample. An average of 90,982 mm^2^ of tissue area was screened to find a positive event. Fluorescent slides mounted with Prolong Gold (Invitrogen) were imaged on an Olympus FV10i confocal microscope using a 60× phase-contrast oil-immersion objective (NA 1.35) and applying a sequential mode to separately capture the fluorescence from the different fluorochromes at an image resolution of 1,024 by 1,024 pixels.

### p24 ELISA and infectivity assay.

p24 concentrations in T-cam2 cells were measured by ELISA according to the manufacturer’s instructions (Innogenetics), and the infectivity of viral particles was determined through the PBMCs’ exposure to T-cam2 cell supernatants for 4 h, with p24 quantification at the indicated time postinfection.

### Analysis of single-cell RNA-sequencing data. (i) Data processing.

Single-cell RNA-sequencing data from human adult testicular cells (85) were recovered from the ReproGenomics Viewer (86, 87) and processed using the AMEN software (88). Briefly, out of the 23,109 genes measured in this experiment, only those with an average expression level [log_2_(count + 1)] of at least 1 in at least one cell population and a fold change of at least 2 between at least two cell populations were considered being differentially expressed. Selected genes were further classified according to their peak expression in testicular macrophages (Cell population tMϕ in the initial publication of reference 85), in Sertoli cells, in spermatogonia (cell populations SSC1, SSC2, differentiating and differentiated SPG in the original publication), in primary or secondary spermatocytes (cell populations L1, L2, L3, Z, P, D, and SPC7 in the original publication), or in round or elongated spermatids (cell populations S1 to S4 in the original publication). The consistency of the results was controlled by comparing expression profiles of all selected genes to those obtained by Hermann et al. (89).

### (ii) Enrichment analysis.

A list of known factors involved in HIV life cycle postentry was retrieved from the literature (Table S1). These were annotated as “Early Co-factors” (79 genes), “Late Co-factors” (28 genes), “Sensors” (15 genes), “Early Inhibitors” (28 genes), or “Late Inhibitors” (185 genes) according to their demonstrated roles. The overrepresentation and underrepresentation of these factors among clusters of differentially expressed genes were then evaluated with a hypergeometric test, while the false-discovery rate was controlled by adjusting the resulting *P* values using the Benjamini and Hochberg method (90).

### Statistical analysis.

Statistical analyses were performed using commercially available software (GraphPad Prism 6; GraphPad Software, Inc., La Jolla, CA). Data were analyzed with the nonparametric Friedman-Dunn's test or Mann-Whitney test, as indicated in the figure legends. Values were considered significant when *P* was <0.05. Statistical analyses performed on single-cell RNA-sequencing data are described above in the relevant section.

## Supplementary Material

Supplemental file 1

Supplemental file 2
