# Supplementary material for: Potential for Virus Endogenization in Humans through Testicular Germ Cell Infection: the Case of HIV
Source: J Virol. 2020 Nov 23;94(24):e01145-20. doi: 10.1128/JVI.01145-20 (PMC7925188; doi:10.1128/JVI.01145-20)
Supplement: Supplemental file 2 [file JVI.01145-20-s0002.pdf]

SUPPLEMENTAL INFORMATIONS

Table S1 List of HIV replication modulating factors expressed by human testicular macrophages and germ cells as detected in single-cell RNA-sequencing data.

A searchable excel file containing various information on 335 factors involved in HIV life cycle is provided, including: the Gene Name (column A), the Entrez Gene ID (column B), the Type of Factor (e.g. “Early inhibitor”; Column C), the Step/Process they affect (e.g. “Nuclear import”; Column D) and the corresponding Pubmed ID(s) (Column E). Subsequent column indicate the Expression Cluster to which they belong (e.g. C1-Macrophages; Column F), according to single-cell RNA-sequencing data from Wang et al. (84). Finally, columns G to V provide the corresponding average gene expression values (log-2 transformed) in each testicular cell population and are color-coded according to low (blue) or high (red) expression levels.
